# Phase-locked mutants of *Mycoplasma agalactiae*: defining the molecular switch of high-frequency Vpma antigenic variation

**DOI:** 10.1111/j.1365-2958.2007.06103.x

**Published:** 2008-01-30

**Authors:** Rohini Chopra-Dewasthaly, Christine Citti, Michelle D Glew, Martina Zimmermann, Renate Rosengarten, Wolfgang Jechlinger

**Affiliations:** Institute of Bacteriology, Mycology and Hygiene, Department of Pathobiology, University of Veterinary Medicine Vienna Veterinärplatz 1, A-1210 Vienna, Austria

## Abstract

*Mycoplasma agalactiae*, an important pathogen of small ruminants, exhibits antigenic diversity by switching the expression of multiple surface lipoproteins called Vpmas (Variable proteins of *M. agalactiae*). Although phase variation has been shown to play important roles in many host–pathogen interactions, the biological significance and the mechanism of Vpma oscillations remain largely unclear. Here, we demonstrate that all six Vpma proteins are expressed in the type strain PG2 and all undergo phase variation at an unusually high frequency. Furthermore, targeted gene disruption of the *xer1* gene encoding a putative site-specific recombinase adjacent to the *vpma* locus was accomplished via homologous recombination using a replicon-based vector. Inactivation of *xer1* abolished further Vpma switching and the ‘phase-locked’ mutants (PLMs) continued to steadily express only a single Vpma product. Complementation of the wild-type *xer1* gene in PLMs restored Vpma phase variation thereby proving that Xer1 is essential for *vpma* inversions. The study is not only instrumental in enhancing our ability to understand the role of Vpmas in *M. agalactiae* infections but also provides useful molecular approaches to study potential disease factors in other ‘difficult-to-manipulate’ mycoplasmas.

## Introduction

Reversible high-frequency changes of bacterial cell surfaces via phase variation are thought to increase resistance against host defences or enhance survival under stressful environmental conditions (Henderson *et al*., 1999; van der Woude and Baumler, 2004). Wall-less bacteria of the *Mycoplasma* genus include the smallest, self-replicating prokaryotes and have lost many biosynthetic pathways during their evolution which reflects their fastidious lifestyles. However, despite a reduced genome, many species possess large multigene families devoted to surface variation and are successful pathogens of immunocompetent hosts (Citti *et al*., 2005). The presence of such elaborate phase-variable systems in these minimalist organisms highlights their significance in pathogenesis but their precise functions are rarely understood (Citti *et al*., 1997; Denison *et al*., 2005).

The ruminant pathogen *Mycoplasma agalactiae* exhibits surface diversity through abundant and variable expression of surface lipoproteins (Vpmas) encoded by a multigene family (Glew *et al*., 2000). Although genetic analysis has revealed the presence of six single-copy *vpma* genes (*vpmaU–Z*) clustered in the type strain PG2, the expression and variability of only two Vpma proteins, namely VpmaU and VpmaY, have been established so far. Sequence analyses showed that each mature Vpma is composed of both unique and conserved amino acid regions that can be shared between two or more *vpma* gene products. (Glew *et al*., 2002). The 5′ untranslated regions and those encoding the signal peptide are conserved within the *vpma* gene family and share a high identity to the equivalent regions of *vsp* genes forming a similar multigene phase-variable system in the bovine pathogen *Mycoplasma bovis* (Lysnyansky *et al*., 1999). Both *vsp* and *vpma* genes contain repeated sequences, exhibit the same lipoprotein cleavage motif (AAKC) and encode similar short cytadherence epitopes (Glew *et al*., 2002). Due to these similarities and the very close phylogenetic relationship between *M. agalactiae* and *M. bovis* (Askaa and Erno, 1976; Pettersson *et al*., 1996), it is likely that the *vpma* and *vsp* loci were inherited from a common ancestor. However, beyond the highly conserved 5′ untranslated and N-terminal regions, the *vsp* and *vpma* genes share no significant homology with each other and their respective coding sequences being very different from one another might have later evolved independently as two separate systems to accommodate different host specificities (Glew *et al*., 2000). Nevertheless, as both these ruminant pathogens induce similar clinical signs (mastitis, pneumoniae and arthritis) in their respective hosts, it is speculated that the two homologous systems, Vpmas and Vsps, might play similar roles during the disease process (Glew *et al*., 2000). Compared with the 13 *vsp* genes described in the *vsp* locus of *M. bovis* type strain PG45 (Lysnyansky *et al*., 1999), the *vpma* repertoire described in the type strain PG2 is much smaller and offers an opportunity to study the mechanism and pathogenicity potential of such multigene antigenic variations. The knowledge gained thereof might provide important clues to understand *M. bovis* infections which cause major economic losses worldwide (Nicholas and Ayling, 2003).

The Vpma and Vsp systems, as well as the Vsa system of the murine pathogen *Mycoplasma pulmonis*, which also belongs to the same *Mycoplasma hominis* phylogenetic cluster (Pettersson *et al*., 2000), represent remarkable examples in terms of the mechanism governing their phase variations. The mechanism governing Vpma, Vsp and Vsa phase variations is thought to be driven by site-specific DNA inversions which either link the ORF of a silent gene to a unique active promoter, as observed in *vpma* and *vsp* genes of *M. agalactiae* and *M. bovis* (Lysnyansky *et al*., 2001; Glew *et al*., 2002; Flitman-Tene *et al*., 2003), respectively, or juxtapose a DNA sequence containing the promoter, ribosome binding site and first 714 nucleotides of the *vsa* coding region in front of the 3′ end of a previously silent *vsa* gene in *M. pulmonis* (Bhugra *et al*., 1995; Shen *et al.*, 2000). Among mycoplasmas, another example where phase variation in a lipoprotein gene family has been described to be mediated by site-specific DNA inversions is the P35 or Mpl family of *Mycoplasma penetrans*. However, most of the 38 *mpl* genes are thought to be independently switched ON↔OFF by DNA inversions occurring in the adjacent invertible promoter sequences and not by juxtaposition of a single promoter sequence within the multigene locus (Horino *et al*., 2003). Another difference is the distribution of the *mpl* gene clusters at different chromosomal loci of *M. penetrans,* which is very similar to the phase-variable systems of certain *Bacteroides* species, where genes encoding capsular polysaccharides and surface glycoproteins are not only distributed throughout the chromosome, but are also similarly switched ON↔OFF by promoter inversions brought about by recombinases having a global action on several different loci (Coyne *et al*., 2003; Fletcher *et al*., 2007; Roche-Hakansson *et al*., 2007).

The presence of sequences resembling recombinase genes, designated *xer1*, *mbr* and *hvsR*, showing high homology to the members of the large protein family of lambda integrases or tyrosine recombinases, has been documented in the vicinity of the *vpma*, *vsp* and *vsa* loci respectively (Glew *et al*., 2002; Ron *et al*., 2002). The Xer1 and Mbr recombinase sequences and their proposed recombination sites located in the 5′ untranslated regions of the genes found in their respective multigene loci share considerable homology (Glew *et al*., 2002; Ron *et al*., 2002). On the other hand, HvsR shows less similarity to Mbr and Xer1, and its known target sites show no homology to the equivalent regions in the *vsp* and *vpma* loci (Glew *et al*., 2002; Ron *et al*., 2002; Sitaraman *et al*., 2002). Among these recombinases, HvsR is the only one whose role in mediating site-specific rearrangements has been experimentally demonstrated (Sitaraman *et al*., 2002), whereas the role of Xer1 and Mbr in controlling similar DNA inversions is predictive and has yet to be proven.

*Mycoplasma pulmonis* also harbours a family of phase-variable restriction and modification enzymes encoded by the variable *hsd* locus (Dybvig *et al*., 1998) that was also found to undergo DNA rearrangements regulated by HvsR. Interestingly, a similar *hsd* locus also exists in *M. agalactiae* but includes an integrase like gene (*int*) (Sirand-Pugnet *et al*., 2007). As *M. pulmonis* is phylogenetically related to *M. agalactiae*, the two phase-variable systems might have evolved from a common ancestor, leaving an intriguing possibility that *vpma*-specific recombinations might be controlled by the Int recombinase, either completely, or in addition to the predicted Xer1 recombinase encoded in the *vpma* locus. In this given scenario, the Xer1 recombinase, like HvsR, could also be predicted to have dual substrate specificity recognizing two distinct recombination sites, and catalyse DNA inversions at both the *vpma* and *hsd* loci of *M. agalactiae.*

Another significant feature of the *vpma* gene locus is the presence of a homopolymeric tract of multiple thymidine residues immediately upstream of the −10 region of the unique *vpma* promoter (Flitman-Tene *et al*., 2003). Such homo- or heteropolymeric tracts are known to undergo frequent and reversible changes in the number of nucleotides via slipped-strand mispairing and lead to variation of cell surface proteins in many mycoplasma species (Citti *et al*., 2005). For instance, the insertion or deletion of nucleotides in the spacer region between the −35 and −10 region has been shown to turn ON or OFF the transcription of the *vlp*, *maa2* and *vmm* genes of *Mycoplasma hyorhinis*, *Mycoplasma arthritidis* and *Mycoplasma mycoides* respectively (Citti and Wise, 1995; Washburn *et al*., 1998; Persson *et al*., 2002). Although site-specific DNA recombination has been proposed as the mechanism underlying Vpma phase variation (Glew *et al*., 2002), slipped-strand mispairing might also be operational in *M. agalactiae* and provide it with another mode of eliciting surface diversity by turning ON or OFF the single promoter present in the *vpma* locus.

The present work was undertaken to investigate the nature of molecular switches involved in Vpma antigenic variations, and to define the recombinase enzyme that controls the expression and variability of all or some *vpma* genes via site-specific DNA inversions within the *vpma* multigene locus. One main impediment in this task was the difficulty in isolating or distinguishing one Vpma variant from another. Therefore, our objectives towards understanding the Vpma system were first to develop specific serological reagents to individually monitor the expression and switching frequency of the six Vpma proteins of PG2, and second to construct ‘phase-locked’ mutants (PLMs) of *M. agalactiae* that constitutively express only a single Vpma protein. Even though the involvement of the *int* gene (MAG5690) in regulating *vpma* inversions could not be ruled out, we first opted for disrupting the *xer1* gene due to its close proximity to the *vpma* locus that has been characterized as a pathogenicity island-like locus (Glew *et al*., 2002) and hence, is likely to carry its own recombinase (Hacker *et al*., 1997). One strategy to inactivate the *xer1* gene in *M. agalactiae* could be to use transposon mutagenesis as done for inactivation of *hvsR* in *M. pulmonis* (Sitaraman *et al*., 2002). However, in order to avoid the possibility of mutant instability associated with transposon mutagenesis, we established a disruption strategy based on homologous recombination (HR). As targeted gene disruptions via HR using classical ‘suicide vectors’ are a very rare phenomenon in mycoplasmas (Dybvig and Woodard, 1992; Dhandayuthapani *et al*., 1999; Markham *et al*., 2003; Burgos *et al*., 2007), we decided to use an *M. agalactiae oriC* plasmid (Chopra-Dewasthaly *et al*., 2005a) to introduce a partial *xer1* gene into PG2 to increase the likelihood of a *xer1* disruptive HR event. Such *oriC* vectors have been successfully used to obtain specific gene disruptants in two mollicutes so far, *Spiroplasma citri* (Duret *et al*., 1999) and *Mycoplasma capricolum* ssp. *capricolum* (Janis *et al*., 2005).

In this study, disruption of *xer1* is demonstrated to abrogate subsequent DNA rearrangements within the *vpma* locus and lead to the generation of PLMs proving that *xer1* is essential for Vpma antigenic switches in *M. agalactiae*. Also, through the use of specific polyclonal antibodies (pAbs) raised against all six Vpma proteins we show that all six Vpmas are expressed within a population of *M. agalactiae* type strain PG2, that this expression is on the surface of the cells, and that all Vpmas exhibit an unexpectedly high frequency of phase variation which was underestimated in previous studies (Glew *et al*., 2000). Comparative sequence analyses of the *vpma* loci of the two PLMs with the published sequence of the clonal variant 55-5 further supports the role of Xer1 in the mechanisms of recombination.

## Results

### All six Vpma proteins are expressed in *M. agalactiae* type strain PG2 and show a remarkably high frequency of phase variation

**Fig. 1:**
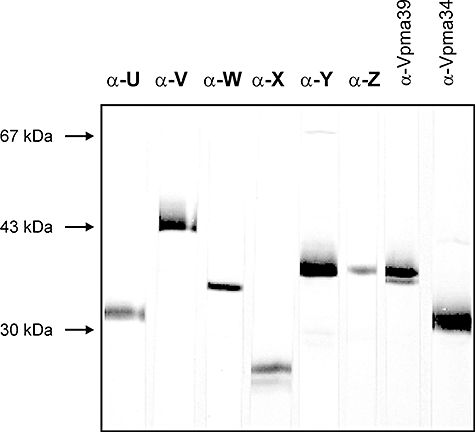
Western blot analyses of Triton X-114 phase material from *M. agalactiae* type strain PG2 using pAbs α-U to α-Z (as described in Table 1). Rabbit pAbs α-Vpma39 and α-Vpma34, previously raised against denatured VpmaY and VpmaU epitopes (Glew *et al*., 2000), respectively, served as positive controls. Protein size standards are indicated in the left margin.

In a previous study, expression and phase variation of only two out of the six *vpma* genes encoded by the PG2 type strain was demonstrated using monoclonal antibodies (mAbs) or VpmaU and VpmaY rabbit antisera (Glew *et al*., 2000; 2002). To further assess the expression profiles and antigenic variation of the entire PG2 *vpma* locus, sequences unique to each of the six *vpma* genes were individually expressed as MBP fusion proteins and used to raise Vpma-specific rabbit pAbs (Table 1, Fig. S1). These antisera identified distinct products in PG2 when subjected to Western blot analysis (Fig. 1). For all Vpma proteins, the observed molecular weight was generally slightly higher than that calculated from their respective sequences (Table 1, Fig. 1). As shown for other mycoplasma proteins (Bhugra *et al*., 1995), this might be due to the presence of repetitive motifs in each Vpma. As expected, α-Y and α-U pAbs recognized the same-sized antigens as identified by the rabbit anti-VpmaY and anti-VpmaU sera obtained in previous studies (Glew *et al*., 2000; 2002).

**Table 1:** Vpma MBP fusion proteins and the corresponding anti-Vpma polyclonal antibodies raised in rabbits.

Vpmas	Primers^a^	Corresponding fusion proteins	Corresponding pAbs	Size of Vpmas (kDa)^b^
VpmaU	U2F/U2R	FP-U	α-U	23.2
VpmaV	C1F/C1R	FP-V	α-V	35
VpmaW	D1F/D1BR	FP-W	α-W	33.1
VpmaX	X1F/X1R	FP-X	α-X	22.4
VpmaY	Y3F/Y3R	FP-Y	α-Y	35.2
VpmaZ	Z2F/Z1R	FP-Z	α-Z	34.2

a Primers used for amplifying and cloning of unique regions for MBP fusion protein production (Table S1 and Fig. S1).

b Calculated for the mature protein (without the signal peptide) based on the individual gene sequences determined for clone 55-5 that expressed VpmaY (Glew *et al*., 2002).

**Fig. 2:**
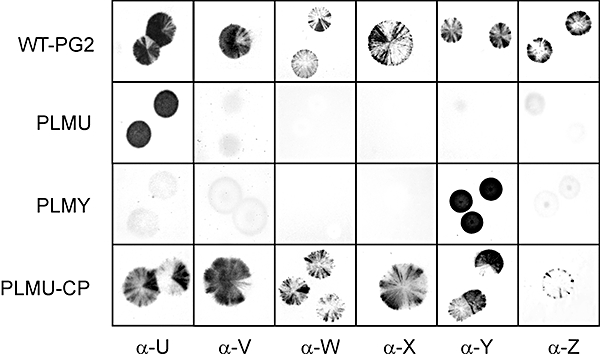
Colony immunoblot analysis of *M. agalactiae* type strain PG2 and its derivative mutants using the six Vpma-specific pAbs recognizing specific surface exposed epitopes. PLMU and PLMY represent the two *xer1*-disrupted PLMs expressing exclusively VpmaU and VpmaY respectively. WT-PG2 and PLMU-CP representing the *xer1*-complemented PLMU show sectorial staining phenotype with all Vpma-specific pAbs reflecting high frequency Vpma phase variations. Designations of Vpma-specific pAbs as used for each column, and as described in Table 1, are indicated at the bottom.

Colony immunoblotting revealed positive, negative and sectorial staining with all six anti-Vpma pAbs reflecting the surface exposure, as well as the hypervariability of all target epitopes in the PG2 strain (Fig. 2, row 1).

### Targeted disruption of the *xer1* gene via HR leads to the isolation of Vpma PLMs

Construction of Vpma PLMs was undertaken to assess whether the *xer1* gene located adjacent to the *vpma* locus controls Vpma phase variation by mediating *vpma*-specific DNA recombination. As attempts to disrupt the *xer1* gene via HR using a suicide (non-replicative) vector repeatedly failed, an alternate strategy was developed. For this purpose, an internal portion of *xer1* was cloned into the replicative *M. agalactiae oriC* vector pMM21-7 that carries the *tetM* gene for tetracycline resistance (Tet^R^) (Chopra-Dewasthaly *et al*., 2005a). The resulting plasmid pR3 was expected to replicate in PG2 thereby increasing the occurrence of otherwise rare HR events. To enrich for rare *xer1* disruptions, the mixture of transformants was passaged in selective media at least five times, followed by two to five passages in non-selective media. The presence of *xer1* disruptants in the transformed population was assessed by a PCR assay designed specifically to detect a 2.0 kb amplicon corresponding to pR3 integration at the *xer1* locus (Fig. S2). PCR-positive transformant mixture passages were freshly grown and subjected to colony immunoblot analyses using pAb α-U. If Xer1 was the enzyme that indeed catalysed *vpma* gene inversions, its disruption was anticipated to abrogate Vpma phase variation and to result in a non-sectored phenotype when immunostained with a particular anti-Vpma pAb. Consequently, non-sectored VpmaU colonies were then picked and screened again by PCR to detect *xer1* disruption. PCR-positive clones were then subjected to three successive rounds of colony purification by colony immunoblot staining and passaging in liquid medium to obtain a rigorously clonal culture expressing VpmaU referred to as PLMU. As illustrated in Fig. 2 (row 2, column 1), the PLMU mutant gave a homogenously positive colony staining phenotype with pAb α-U and showed no negative or sectorial immunostaining. Further phenotypic verification of the selected PLMU clone was performed by subjecting it to colony immunostaining with the other five Vpma-specific antisera, namely α-V, α-Y, α-Z, α-X and α-W, producing a negative phenotype (Fig. 2, row 2, columns 2–6) thereby proving the unavailability of the corresponding epitopes on its cell surface.

**Fig. 3:**
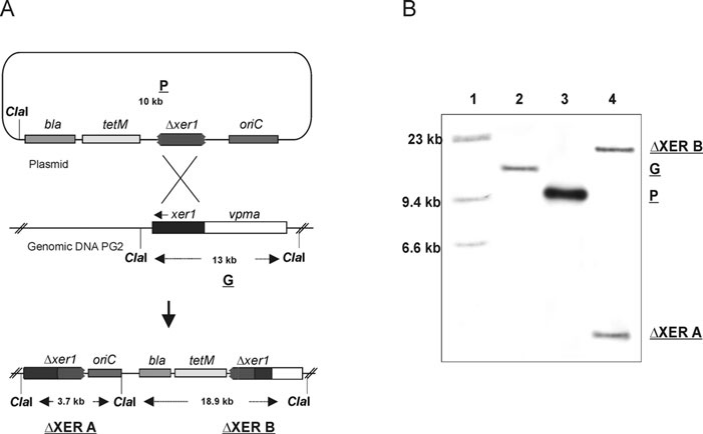
Disruption of *xer1* recombinase in *M. agalactiae*. A. Schematic representation of the integration of disruption plasmid pR3 (P) into the genomic DNA of type strain PG2 to generate the PLMU and PLMY clones. A 13 kb ClaI fragment of PG2 (G) consists of the known 9.6 kb *vpma* locus including the six *vpma* genes (white region) and the complete *xer1* gene (black shaded region). A single putative homologous recombination event between the partial *xer1* sequence carried by the plasmid pR3 and the chromosomal *xer1* region is represented by crossed lines. This crossing over would lead to the integration of pR3 into the chromosome and would segregate the *xer1* region onto two ClaI fragments ΔXERA and ΔXERB. B. Southern blot hybridization showing the localization of pR3 at the chromosomal *xer1* locus of *M. agalactiae* PG2. ClaI-digested DNA of *xer1* disruptant (PLMU or PLMY) (lane 4), disruption plasmid pR3 (lane 3) and wt strain PG2 (lane 2) were probed with a *xer1*-specific DIG-labelled fragment. λ-HindIII DNA size marker (lane 1).

Southern blot hybridization using a *xer1*-specific probe demonstrated that the integration of plasmid pR3 did in fact occur by HR at the chromosomal *xer1* locus (Fig. 3). The chromosomal *xer1* is carried in wild-type (wt) *M. agalactiae* PG2 on a fragment of 13 kb (Glew *et al*., 2002) (depicted as G in Fig. 3) and known to contain the 9.6 kb *vpma* locus (Glew *et al*., 2002; Sirand-Pugnet *et al*., 2007) (Fig. 3A). As the disruption plasmid, pR3, contains a unique ClaI site, plasmid integration via a single HR event at the chromosomal *xer1* locus was expected to result in duplication of the partial *xer1* sequence that would be segregated onto two ClaI fragments (Fig. 3). As predicted, the 13 kb fragment is shown to be absent in the *xer1* disruptant (PLM), and instead, displays two hybridization signals corresponding to (i) a 3.7 kb fragment carrying the plasmid *oriC* region and part of the C-terminal coding region of *xer1* (depicted as ΔXERA in Fig. 3), and (ii) a 18.9 kb fragment carrying the *bla* and *tetM* plasmid sequences, and the N-terminal region of *xer1*, followed by the *vpma* genes (depicted as ΔXERB in Fig. 3). The 10 kb fragment corresponding to the linearized free replicating plasmid (P) was absent in PG2 and in *xer1*-disrupted PLM (Fig. 3B).

**Fig. 4:**
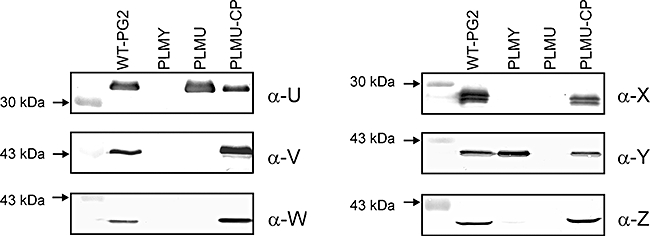
Comparative Western blot analysis of whole-cell extracts of *M. agalactiae* type strain PG2 (WT-PG2), two PLMs (PLMY and PLMU) and *xer1*-complemented PLMU (PLMU-CP) using six different Vpma-specific pAbs as described in Table 1. Designations of individual pAbs used for each Western blot are indicated in the right margins of each panel whereas relevant protein size standards are shown on the left margins.

Western blot analysis using whole-cell extracts of PLMU confirmed the results of colony immunoblot analysis (Fig. 4). PLMU only reacted with pAb α-U, corresponding to the VpmaU protein, and was not recognized by any of the other five pAbs. Consistent expression of only VpmaU upon 15 successive passages of PLMU, both in the absence and presence of tetracycline selection**,** and the complete lack of any spontaneous reversions or *vpma*-specific rearrangements proved the ‘phase-locked’ VpmaU phenotype in this *xer1* mutant (data not shown). This was in contrast to the wt parental strain PG2 which reacted with all six pAbs, and whose clonal variants expressing VpmaU led to a mixed colony Vpma-staining phenotype (positive, negative and sectorial) with almost all pAbs within two to five *in vitro* passages, and as well showed *vpma*-specific rearrangements observed by Southern blot analysis (data not shown) indicating Vpma phase variation.

To further confirm the role of *xer1* in Vpma phase variation, the same strategy was exploited to independently obtain PLMY using pAb α-Y. This mutant showed a completely positive staining pattern with pAb α-Y and was negative with all the other five antisera (Fig. 2, row 3). Disruption of *xer1* in PLMY was further confirmed by Southern blot analysis as described above. Like PLMU, PLMY was phenotypically and genotypically stable even after several *in vitro* passages (data not shown). Western blot analysis using the whole-cell extracts of PLMY (Fig. 4) supported the results of colony immunoblotting (Fig. 2). PLMY showed a specific product of ∼39 kDa with pAb α-Y as expected and was negative with all other pAbs, except with pAb α-Z which revealed a very faint band corresponding to the presence of VpmaZ in this mutant, although to a very low extent (Fig. 4). However, no positive staining pattern was observed during colony immunoblot analysis of PLMY using α-Z antisera, probably due to the very low expression level of VpmaZ.

### Sequence analyses of the *vpma* gene loci of PLMU and PLMY: gene organization and Rho-independent terminator structures

In order to precisely define the configuration of the *vpma* loci in the PLMY and PLMU mutants, their ClaI-digested genomic DNA were, respectively, self-ligated and used to transform *Escherichia coli* DH10B. Tetracycline- and ampicillin-resistant transformants were isolated and subjected to restriction and PCR analyses (data not shown). Two 18.9 kb recombinant plasmids, designated as pPLMU and pPLMY, carrying the *vpma* loci (8.6 kb) of PLMU and PLMY, respectively, were isolated for further examination.

**Fig. 5:**
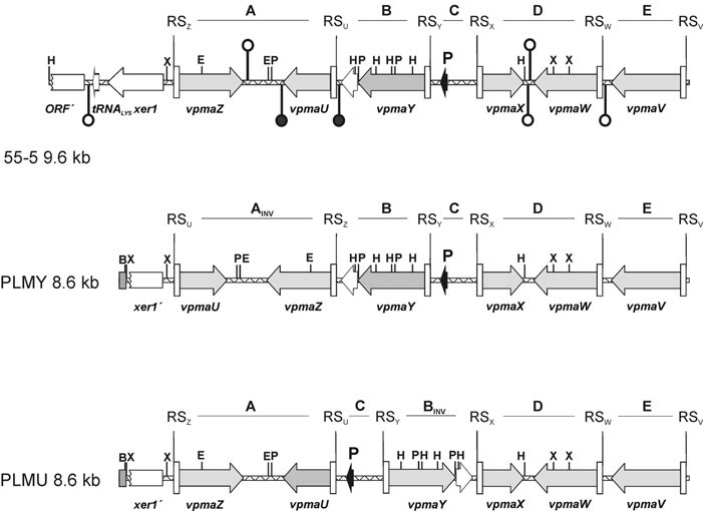
Arrangement of the ORFs, terminator structures and inversions in the *vpma* loci of *M. agalactiae* type strain PG2 clonal variant 55-5, PLMY and PLMU. Grey arrows represent the *vpma* genes, whereby a dark grey arrow denotes the *vpma* gene which is transcribed in the respective clonal variant or PLM. The black arrows indicate the location of the unique promoter (P) in each *vpma* locus. Putative recombination sites are indicated by white rectangles (RS) and denote sequences from −1 to −72 relative to the start codons of the corresponding *vpma* genes. White arrows indicate ORFs other than *vpma* genes, like the intact *xer1* recombinase (*xer1*) and a tRNA (tRNA_LYS_) gene in 55-5, whereas the discontinued white rectangles represent the promoter proximal regions of the disrupted *xer1* genes (*xer*′) in the PLMs and a partial ORF (ORF′) in 55-5. A small grey rectangle indicates sequences derived from the integrated plasmid pR3. Non-translated repeats of the *vpmaY* genes are indicated with a dotted-lined white arrow. Terminator structures are indicated with symbolized hairpins, whereby Rho-independent terminator structures, for which experimental evidence is given, are drawn with a black-filled loop and are white-filled otherwise. Sequence-identical blocks between PLMs and 55-5 are designated A to E and are indicated with _INV_ when inverted compared with 55-5. B, BamHI; E, EcoRI; H, HindIII; P, PstI; X, XbaI.

DNA sequencing of the pPLMY *vpma* locus revealed a *vpma* gene organization similar to that found in the clone 55-5 (Fig. 5), which is known to express VpmaY (Glew *et al*., 2002), and whose entire genome was recently sequenced (Sirand-Pugnet *et al*., 2007). In pPLMY, as the *vpmaZ* gene is located downstream of *vpmaY* and in the same transcriptional orientation, a low level of ‘read-through’ could explain the presence of VpmaZ as background expression in PLMY. In pPLMU, *vpmaZ* is placed downstream of the transcribed *vpmaU* gene, but in the opposite direction (Fig. 5) and thus, PLMU exhibits the sole expression of VpmaU. In an earlier study of Glew *et al*. (2002), Northern blot analyses indicated the presence of a single 1.6 kb *vpmaY*-mRNA in clone 55-5 and a single 1 kb *vpmaU*-mRNA in clonal variant 55-7 that expressed the VpmaU protein. The length of the detected *vpmaY* transcript in 55-5 suggests the presence of a termination structure immediately before the *vpmaU* gene. Likewise, a termination structure must be present shortly after *vpmaU* based on the data pertaining to 55-7. Using the individual Vpma-specific pAbs, we could detect a very low amount of VpmaZ protein in PLMY corresponding to the *vpmaY* gene located downstream of *vpmaY* thereby suggesting a transcriptional control of termination. As mycoplasmas are known to lack the Rho protein (de Hoon *et al*., 2005), we searched for putative Rho-independent transcriptional terminators in the intergenic regions of the *vpma* loci that are characterized by an inverted repeat followed by a stretch of thymidine residues in the primary DNA sequence (Nudler and Gottesman, 2002; de Hoon *et al*., 2005). As described in *Experimental procedures*, we used mathematical models based on (i) the Gibbs free energy (−Δ*G*) of stem-loop formation in the RNA and (ii) the properties of the thymidine stretch in the primary DNA sequence (de Hoon *et al*., 2005) to detect putative terminator structures in the *vpma* locus (Table 2). *In silico* results indicated the presence of such a structure 1560 bp downstream of the *vpmaY* transcription start that is in perfect agreement with the mRNA length detected by Northern blot analysis by Glew *et al*. (2002). Similarly, a very strong termination structure was detected just 40 bp downstream of the stop-codon of the *vpmaU* gene and 970 bp downstream of the transcriptional start of the *vpmaU* gene and correlates well with the earlier mRNA studies (Glew *et al*., 2002) (Fig. 5, Table 2). Further analysis of other *vpma* genes also revealed the presence of Rho-independent terminator structures between 40 and 80 nucleotides downstream of the respective stop-codons (Fig. 5, Fig. S3). Although no terminator was found immediately downstream of the *xer1* gene, a strong termination signal was detected just downstream of the tRNA-lys (anti-CTT) gene suggesting the bicistronic organization of these two genes (Fig. 5).

**Table 2:** Characteristics of Rho-independent terminators of the *vpma* locus.

	Hairpin −Δ*G*^a^	Stem length^b^	Loop size^c^	T-stretch No. of Ts	T-stretch score^d^	Decision rule *d*^e^
Ter U	6.3	4	4	12	5.9	5.1
Ter V	13.7	10	5	8	4.8	2.3
Ter W	9.5	8	8	10	5.4	2.8
Ter X	7.5	9	6	11	5.8	3
Ter Y	5.8	7	3	9	5.3	1.7
Ter Z	6.3	6	8	11	5.8	3.1
Ter Xer	13.2	9	5	10	5.2	3.5
Mean	8.9	7.6	5.6	10.1	–	–
*Mycoplasma synoviae*^e^	8.4	7.7	5.1	10.7	–	–
*Escherichia coli*^e^	15.1	10.2	5.2	8.4	–	–
*Bacillus subtilis*^e^	14.9	9.1	5.2	9.3	–	–

a Gibbs free energy of stem-loop formation in kcal/mole at a temperature of 25°C.

b Number of nucleotide pairs in the stem (see Fig. S3).

c Number of nucleotides forming the loop structure (see Fig. S3).

d Calculated according to de Hoon *et al*. (2005) (see *Experimental procedures*).

e Mean values for predicted terminator structures in the genome of the respective bacteria according to de Hoon *et al*. (2005).

The sequences of PLMY and PLMU located between the proposed recombination sites (RS, between position −1 to −72 relative to the start codon of all *vpma* genes) are identical to the published sequences of 55-5 (Glew *et al*., 2002), even though some blocks were inverted as expected (blocks A–E, Fig. 5). The 72 nucleotides of the putative RS of each *vpma* gene (RS_U_–RS_Z_) differ maximally at five positions when aligned, whereas the 21 bp regions between position −51 and −72 exhibit complete sequence identity between all RS as also described by Glew *et al*. (2002) for 55-5. Thus, recombination events must occur in between the nucleotide position −72 and the start codon of the respective *vpma* genes, otherwise the sequences of blocks A–E (Fig. 5) would not be completely identical in 55-5, PLMY and PLMU. Furthermore, the recombination sites remain unaltered during different *vpma* recombinatorial episodes as no nucleotide changes were detected while comparing the sequences with the published 55-5 sequence.

### Complementation of the *xer1* gene in PLMs restores Vpma phase variation

In order to confirm that *xer1* expression is indeed essential to Vpma switching, a complete *xer1* gene along with its putative promoter was introduced into PLMY and PLMU to restore phase variation. Initially, the entire *xer1* gene along with its putative promoter was cloned in Tn*4001*mod (Knudtson and Minion, 1993) and the resultant pR4 vector randomly inserted by transposition in the genome of both PLMs. The same cloning sites of Tn*4001*mod have been previously used in other mycoplasma species for the successful expression of different genes upon chromosomal integration (Hahn *et al*., 1996; Fisseha *et al*., 1999; Dybvig *et al*., 2000; Liu *et al*., 2000; Waldo and Krause, 2006). Although the presence of the *xer1* gene at different chromosomal loci of several gentamicin-resistant (Gent^R^) PLM clones was clearly evident by PCR and Southern analysis (data not shown), it failed to provide functional complementation in terms of restoring Vpma phase variation.

**Fig. 6:**
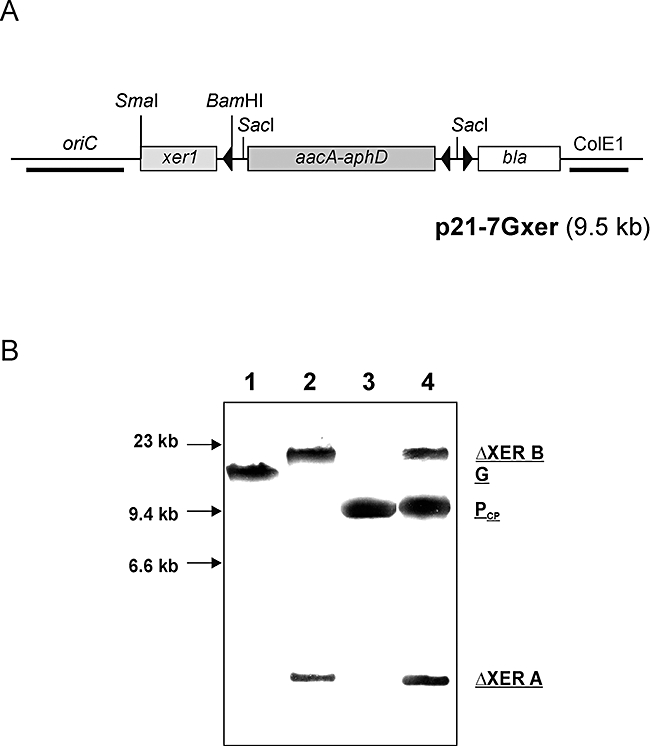
Complementation of the wt *xer1* gene in PLMU. A. Schematic representation of complementation plasmid p21-7Gxer. Restriction sites used for cloning purposes are as indicated and arrowheads represent direction of transcription; ColE1, *E. coli* origin of replication; *bla*, ampicillin resistance gene; *aacA-aphD*, Gent^R^ gene; *oriC*, *M. agalactiae* origin of replication. B. Southern blot analysis of *xer1*-complemented PLMU. ClaI digest of DNA from the complementation clone PLMU-CP (lane 4) is shown in comparison with wt strain PG2 (lane 1), PLMU (lane 2) and complementation plasmid p21-7Gxer (lane 3) after hybridization with a *xer1*-specific DIG-labelled probe. G represents the 13 kb ClaI fragment of *M. agalactiae* type strain PG2 whereas ΔXERA and ΔXERB represent the two ClaI fragments from PLMU (as described in Fig. 3); P_CP_ depicts the 9.5 kb linearized complementation plasmid p21-7Gxer. DNA size standards are indicated in the left margin.

As an alternative strategy, *xer1* along with its putative promoter was cloned in an *oriC* vector carrying the Gent^R^ selection marker to generate the complementation plasmid p21-7Gxer (Fig. 6A), which was transformed into the Tet^R^ mutants PLMU and PLMY. Interestingly, the transformation frequency (TF) of p21-7Gxer in PLMs was considerably low [4.3 × 10^−8^ transformants per colony-forming units (cfu)] compared with the other replicative plasmid pR3 when transformed into PG2 (8 × 10^−6^ transformants per cfu). This implies that the introduction of a third *oriC* fragment into the PLMs may be detrimental to the cells. However, the TF of the complementation plasmid p21-7Gxer into PG2 was also slightly lower (3 × 10^−7^) than pR3, and this might be a consequence of *xer1* overexpression. Also, the p21-7Gxer-complemented PLM transformants sometimes displayed distorted colony morphology upon initial selection on agar plates containing tetracycline and gentamicin. However, subsequent growth and platings showed a normal phenotype without any irregularities. The transformants were confirmed by PCR for the presence of the complete *xer1* gene, the Tet^R^ and Gent^R^ markers (data not shown). Selected positive clones analysed in Southern hybridization using the *xer1*-specific probe clearly demonstrated the presence of plasmid p21-7Gxer as shown for the identically complemented PLMU clone in Fig. 6B (lane 4). Besides the two bands associated with the chromosomal *xer1* disruption in PLMs, the complemented clones depicted an additional hybridization signal corresponding to the ∼9.5 kb free p21-7Gxer plasmid. Chromosomal integration of p21-7Gxer was concluded to be absent as no additional hybridization signals were observed in Southern blot analysis.

Having confirmed the episomal presence of the complementation *xer1* gene copy, selected clones were subjected to colony immunoblot analysis using the six Vpma-specific pAbs to ascertain functional *xer1* complementation in the two PLMs. In Fig. 2 (row 4), the *xer1*-complemented PLMU shows a mixed (positive, negative and sectorial) staining pattern with all pAbs implying that it now exhibits variable surface expression of all Vpma proteins in contrast to its parent *xer1*-disrupted PLMU which shows a positive and complete reaction only with the α-U antisera and no other pAb. Similar results were obtained during Western blot analysis where the *xer1*-complemented PLMU was shown to express each of the six distinct Vpma proteins, similar to the wt PG2 strain (Fig. 4). This is unlike its predecessor, PLMU, which is recognized only with the α-U antiserum. Similar genotypic and phenotypic analyses were also performed with *xer1*-complemented PLMY (data not shown) and the results confirmed that Xer1 is also essential for PLMY regaining Vpma phase variation. PLMs transformed with the complementation plasmid lacking the *xer1* gene (control plasmid p21-7G) were not able to restore phase variation and were phenotypically identical to the parent *xer1*-disrupted PLMs. These data clearly demonstrate that the presence of a functional Xer1 protein in both PLMU and PLMY initiates *vpma*-specific gene inversions that generate cell surface Vpma phase variation comparable to that occurring in the wt PG2 strain.

## Discussion

The study of phase variation and the mechanisms by which it is elicited provides an insight into the processes by which a pathogen is able to survive within varied and complex host niches. Several genetic mechanisms of pathogenic mycoplasmas have been described which enable them to change their antigenic repertoire with an unusually high frequency (Citti *et al*., 2005). Three members of the *M. hominis* cluster, *M. agalactiae*, *M. bovis* and *M. pulmonis*, have developed similar mechanisms of mediating phase variation in their respective *vpma*, *vsp* and *vsa* gene families, where site-specific DNA rearrangements place alternative silent genes downstream of DNA sequences that contain single unique promoters. *M. penetrans*, belonging to the *Mycoplasma pneumoniae* cluster, also exhibits phase variation via site-specific DNA inversions in the *mpl* multigene family that consists of 38 genes. However, the expression of these genes is proposed to be independently switched ON↔OFF by adjacent invertible promoter sequences (Horino *et al*., 2003). Two ORFs (MYPE8180 and MYPE2900), located within the vicinity of the *mpl* genes, have been suggested as candidates for mediating *mpl* recombinatorial events (Horino *et al*., 2003). These recombinases display significant similarities with the Xer1, Mbr and HvsR recombinases of the *vpma*, *vsp* and *vsa* loci respectively (Chambaud *et al*., 2001; Ron *et al*., 2002; Sirand-Pugnet *et al*., 2007). In the same phylogenetic *M. pneumoniae* group, *Ureaplasma parvum* encodes three recombinases (UU222, UU145 and UU529), whose functions have not been proven, but are also closely related to Xer1, Mbr and HvsR (Glass *et al*., 2000). Despite the existing similarity between these recombinases, genes of the respective phase-variable systems do not display any homology to each other, except for the signal sequences of *vsp* and *vpma* genes belonging to two closely related *Mycoplasma* species. This might have been the result of adaptive evolution, either to colonize different host species or to perform different functions during the disease process.

Although the Vpma, Vsp and Vsa antigenic variation systems have been well studied at the DNA level, phenotypically, these proteins have only been observed using immunoblot analysis employing mAbs that recognize more than one member of the same multigene family. For instance, mAb 7.1-2 recognizes all the proteins of the Vsa family (Gumulak-Smith *et al*., 2001), whereas mAb 1E5 and mAb 3B3 recognize several proteins of the Vsp and Vpma family respectively (Bergonier *et al*., 1996; Lysnyansky *et al*., 1999; Glew *et al*., 2000). The switching frequencies calculated on the basis of results obtained with such mAbs are expected to underestimate the rate of variation of these proteins. The calculation of the switching frequency of variable proteins is based on the evaluation of the progeny of a clonal variant which at first expresses only one distinct protein (Rosengarten and Wise, 1990; Glew *et al*., 2002). The phenotype of the progeny of this clonal variant can then be tested for the expression (+) or non-expression (−) of the respective protein via colony immunoblots. By using pAbs specific to each of the six individual Vpma proteins, the switching frequency was observed to be much higher than the earlier estimated frequency of 10^−3^ to 10^−2^ per cell per generation based on the results obtained with *M. agalactiae*-specific mAb 3B3 (Glew *et al*., 2000). In the present study we show that mAb 3B3 also recognizes VpmaX and VpmaW (Fig. S4), in addition to the already reported VpmaY (Glew *et al*., 2000). Rather than expressing a (+) or (−) phenotype for a specific Vpma protein, a vast majority of PG2 colonies exhibited a highly sectored phenotype with these monospecific pAbs, and made it impossible to denote a numerical value for Vpma switching frequency using standard methods. Extrapolating these results to the parallel system of Vsp proteins in *M. bovis*, it is likely that the Vsp switching frequency is also much higher than the earlier reported frequency of 10^−3^ to 10^−2^ per cell per generation (Lysnyansky *et al*., 1996).

In a recent study, the role of Vsa phase variations was investigated in an animal experiment by PCR using the *M. pulmonis* strain CT-AD as inoculum (Denison *et al*., 2005). Although this study provided useful information regarding the avoidance of the host immune system through Vsa phase variation in *M. pulmonis*, similar studies would be impossible using wt *M. agalactiae* strains due to the extremely high frequency of Vpma switching. Phase-variable lipoproteins are abundantly expressed in mycoplasmas but their precise biological significance remains speculative. PLMs could serve as important tools in comprehending the relevance of phase variation of mycoplasma lipoprotein families during *in vitro* and *in vivo* pathogenicity studies.

Disruption of the *xer1* gene led to a Vpma ‘phase-locked’ phenotype in *M. agalactiae* proving that Xer1 recombinase is indeed responsible for the site-specific recombinations occurring within the *vpma* gene locus. The results also demonstrate that the homopolymeric T-tract found upstream of the unique *vpma* promoter does not play any role in generating variation via slipped-strand mispairing as none of the several independently screened PLMY and PLMU colonies ever showed a negative phenotype where the respective VpmaY or VpmaU protein was not expressed (data not shown).

In this study, we have described the presence of Rho-independent terminator structures in the *vpma* locus and have further strengthened the theory of the monocistronic organization of the *vpma* genes. Nevertheless, the hypothesis of a single Vpma protein being expressed exclusively in all clones at all times does not seem to be totally foolproof. The coexpression of VpmaZ in PLMY is not surprising as the terminator of the *vpmaY* gene seems to be the weakest terminator found in the *vpma* locus (lowest *d* value) and the observed ‘read-through’ could be a logical consequence of intermediate termination efficiency (Table 2, Fig. 5 and Fig. S3).

In mycoplasmas, the organization of terminator structures has been proposed to be composed of weaker stem-loop structures but very T-rich T-stretches (de Hoon *et al*., 2005). The obtained values for the stem-loops and the number of T's in the T-stretch correlate nicely between the terminators found in the *vpma* locus of *M. agalactiae* and in the predicted terminators for *Mycoplasma synoviae*, which represents the nearest related mycoplasma species investigated by de Hoon *et al*. (2005) (Table 2, Fig. S3).

Sequence analysis of the *vpma* loci of PLMY, PLMU and clone 55-5 revealed that sequences most distant to the *xer1* gene remained completely unaltered (Fig. 5, blocks D and E) whereas the *xer1* proximal sequences were rearranged (Fig. 5, blocks A–C). Although this might be just a coincidence, it could, as well, be speculated that the Xer1 recombinase functions preferentially *in cis* as witnessed for certain transposases (Altenbuchner and Schmitt, 1983; McFall, 1986; Adams *et al*., 2006), and this would explain the observed predominance of VpmaU, VpmaY and VpmaZ in the PG2 population (data not shown). The failure of *xer1* complementation by transposition may also support the hypothesis that Xer1 acts in a *cis*-like fashion, with RS site preference inversely proportional to the distance of the *xer1* gene from its target RS sequence. Attempts to restore phase variation in PLMs by transposition, using a transposon containing a wt *xer1* gene with its own putative promoter, were not successful, even when analysed in many different mutants that represented insertions at different chromosomal loci. Thus, the proximity of the *xer1* gene to the *vpma* locus might be an important factor for its functional activity. However, the introduction of the *xer1* gene via a multiple copy *oriC* plasmid restored phase variation, which may be due to the overproduction of the Xer1 protein that compensates for the necessity of *xer1* gene to be located adjacent to the *vpma* locus.

Although complementation of the wt *xer1* gene restored phase variation, we cannot totally rule out the involvement of other factors, including global regulatory proteins, in controlling *vpma* gene inversions as observed in other cases of bacterial phase variation involving gene inversions (Henderson *et al*., 1999). It would be interesting to know if Vpma phase variation events are random and then selected by environmental factors, or are regulated by the pathogen through other, as yet, unidentified factors and regulators that might be operating only inside the host. Taking into consideration the work by Sitaraman *et al*. (2002) where HvsR was shown to control the *vsa,* as well as the *hsd* phase-variable loci, it would be interesting to know if the Xer1 recombinase of *M. agalactiae* plays any role in regulating the *hsd* locus in this species, especially as an integrase-like *int* gene (MAG5690) is present within its *hsd* locus (Sirand-Pugnet *et al*., 2007).

This study demonstrates for the first time that all six *vpma* genes of the *vpma* multigene family are expressed on the cell surface of variants comprising the *M. agalactiae* type strain PG2. To our knowledge, this has not been shown for all members of the *vsa* and *vsp* multigene families of *M. pulmonis* and *M. bovis*, respectively, which are close phylogenetic relatives of *M. agalactiae*. For instance, in a study conducted by Denison *et al*. (2005), 94% of the randomly checked clones from the CT-AD strain of *M. pulmonis* were found to express VsaG and 6% VsaH, whereas the other five Vsa proteins were probably expressed, if at all, at levels too low to be detected by the applied PCR analysis. The anti-Vpma pAbs developed in this study will serve as important reagents to specifically monitor the expression of each individual Vpma product during future *in vitro* and *in vivo* studies. Additionally, they could also help to assess the potential of Vpmas to be used as serological tools for early detection of infected animals. In general, mycoplasma lipoproteins are strongly immunogenic in their natural hosts and despite their intrinsic variability they are known to induce an early and lasting humoral immune response (Citti *et al*., 2005). Hence these Vpma-specific antisera have the potential to be developed into efficient disease markers for the control and eradication of *M. agalactiae* infections.

To sum up, this study is a significant step in mycoplasma genetics as it describes the first targeted gene inactivation through HR in *M. agalactiae* and provides novel insights into the mechanisms of antigenic variation in *M. agalactiae*. Generation of PLMs offers a novel concept in elucidating the role of mycoplasma phase-variable lipoproteins in host–pathogen interactions. Overall, the findings of this study are anticipated to improve our understanding of the mechanisms which enable mycoplasmas to cause chronic and difficult-to-eradicate infections.

## Experimental procedures

### Bacterial strains and culture conditions

This study was carried out on *M. agalactiae* type strain PG2 (Solsona *et al*., 1996) grown at 37°C in modified Aluotto and SP-4 medium supplemented with penicillin, pyruvate and phenol red as described previously (Chopra-Dewasthaly *et al*., 2005b). Mycoplasma transformants were selected on SP-4 medium containing 1% Difco^TM^ Agar Noble and 2 μg ml^−1^ tetracycline or/and 50 μg ml^−1^ gentamicin as appropriate. Cloning and amplification of recombinant plasmids was carried out in *E. coli* DH10B (Invitrogen) grown at 37°C in standard Luria–Bertani medium (Sambrook *et al*., 1989). The latter was supplemented with 50 μg ml^−1^ ampicillin and 10 μg ml^−1^ tetracycline or 7 μg ml^−1^ gentamicin as per selection requirements.

### Raising Vpma-specific pAbs

Rabbit antisera specific to the six Vpma proteins were raised as follows. The unique and specific DNA sequences corresponding to each of the six *vpma* genes were cloned in frame to the *malE* gene in a pMAL^TM^-c2 vector system to obtain the respective MBP fusion proteins (see Table 1 and Table S1, Fig. S1) according to the manufacturer's instructions (New England Biolabs). Protein expression was carried out in *E. coli* TB1 (New England Biolabs) upon induction of the *lacZ* promoter located upstream of the *malE* gene with 3 mM isopropyl-β-d-thiogalactopyranoside (IPTG). Whole-cell extracts were sonicated and centrifuged and the cytoplasmic protein fractions were either purified via affinity chromatography (for FP-U and FP-W) using an amylose column, or loaded on an 8% non-denaturing polyacrylamide gel (for FP-V, -Z, -X and -Y) to excise the appropriate bands after electrophoresis and negative staining with zinc acetate (negative staining kit, Bio-Rad). After destaining, the bands were shredded into smaller pieces and frozen overnight before lyophilization. The lyophilized proteins were re-suspended in phosphate-buffered saline (PBS) and quantified with the BCA kit (Pierce, Rockford, IL). About 100 μg of each purified protein was injected into two different New Zealand White rabbits as described previously (Glew *et al*., 2000). The resultant monospecific antisera were then analysed in Western blots and colony immunoblots using type strain PG2, clonal variant 55-5 (expressing Vpma Y; Glew *et al.*, 2000) and similar pBAD expressed His-tagged fusion proteins of VpmaV, VpmaU, VpmaX and VpmaY. The latter were made by excising the *vpma*-specific inserts from the corresponding pMAL recombinant clones by EcoRI and HindIII digestion and cloning them at the same sites of the pBAD/His B vector (Invitrogen). The working dilutions for the six rabbit pAbs were standardized against PG2 whole-cell lysates in Western blots and colony immunoblots (α-U, 1:400; α-V, 1:100; α-W, 1:600; α-X, 1:200–1800; α-Y, 1:500–2000; α-Z, 1:100–600).

### Colony immunoblot analysis

Protran® nitrocellulose membranes (Schleicher and Schuell, Germany) with a pore size of 0.2 μm were placed on freshly grown mycoplasma colonies on the surface of agar plates for about 3–5 min before detaching and drying them at room temperature (RT). The membranes were rinsed two to three times in TS buffer (10 mM Tris, 154 mM NaCl, pH 7.4) before an overnight incubation at 4°C in Vpma-specific, appropriately diluted antisera in TS buffer. Membranes were then washed three times (for 10–15 min each) in TS buffer containing 0.05% Tween® 20 (Roth) and then incubated for a minimum of 1 h at RT in 1:2000 dilution of swine anti-rabbit IgG conjugated to horseradish peroxidase (DakoCytomation, Denmark). After three washes (10 min each) in TS Buffer, the colony blots were developed for 15–30 min in 4-chloro-1-naphthol (Bio-Rad) and hydrogen peroxide. The reaction was stopped by washing the blots in water. Colony immunoblots made with mAb 3B3 (1:300) (Bergonier *et al*., 1996) were similarly developed after incubating with a goat anti-mouse IgG (1:1000) procured from Jackson ImmunoResearch, USA. All antibody dilutions were made in TS buffer and the blots were agitated on a rocking platform during all incubation and washing steps. The blots were then viewed and photographed using a Nikon SMZ-U stereomicroscope.

### Western blotting

Vpma antigenic phenotypes were analysed by standard sodium dodecylsulphate-polyacrylamide gel electrophoresis (SDS-PAGE) using whole-cell extracts or Triton X-114 (Sigma) fractions as described elsewhere (Citti and Wise, 1995) in 10–12% polyacrylamide gels containing 3% (w/v) urea. Samples were treated at 95°C for 5 min under reducing conditions. Separated proteins were electrophoretically transferred to Protran® membranes using blotting buffer (48 mM Tris, 39 mM Glycine, 0.037% SDS and 20% Methanol) and immunostained by the same protocol as described for colony immunoblots, except that when required sheep anti-*M. agalactiae* serum (PAL) (Glew *et al*., 2000) was used at a concentration of 1:200 and developed similarly after incubation with rabbit anti-sheep IgG (1:2000) obtained from DakoCytomation, Denmark.

### DNA isolation, manipulation and Southern hybridization

Standard molecular biology procedures were used (Sambrook *et al*., 1989) except when stated otherwise. Plasmid DNA was isolated from *E. coli* using E.Z.N.A.® Plasmid Miniprep Kit (Peqlab Biotechnologie GmbH). Mycoplasma genomic DNA was isolated by QIAamp® DNA Mini Kit (Qiagen). Restriction endonucleases (Promega or New England Biolabs) and T4 DNA ligase (Roche) were used according to the manufacturers' instructions. QIAquick® PCR Purification and Gel Extraction Kits (Qiagen) were used for routine DNA purification during various cloning steps. Southern blot and hybridization techniques using DIG-labelling system (Roche) have been described previously (Chopra-Dewasthaly *et al*., 2005a) and were performed on ClaI-digested DNA using a DIG-labelled probe corresponding to the 513 bp partial *xer1* PCR product (described below).

### PCR amplifications

All DNA amplifications were carried out on a Perkin Elmer GeneAmp thermal cycler using GoTaq® Flexi DNA Polymerase (Promega) in 1× buffer supplied by the manufacturer in the presence of 200 μM dNTPs and 1.4 μM of specific primers.

The partial *xer1* gene was amplified using XerR and XerS primers with PG2 genomic DNA template in the presence of 2.5 mM MgCl_2_. An initial denaturation step of 7 min at 95°C was followed by 30 cycles of 95°C for 43 s, 56°C for 43 s and 72°C for 43 s, and terminated with a 7 min cycle at 72°C to yield a 513 bp amplicon.

Detection of *xer1* disruption by integration of plasmid pR3 via HR at the chromosomal *xer1* site was carried out in 25 μl reactions using 2–4 μl of crude DNA extracts (Chopra-Dewasthaly *et al*., 2005b), 2 mM MgCl_2_ and primers T3ISLrev and RecendET28. Cycling parameters consisted of 1 cycle of 5 min at 94°C, 30 cycles of 1 min at 94°C, 1 min at 56°C and 2 min at 72°C and a concluding cycle of 5 min at 72°C. The presence of a *xer1* disruption event was identified by the presence of a 2 kb product after PCR.

The 2.5 kb fragment containing the Gent^R^ determinant was amplified from plasmid pISM2062 (Knudtson and Minion, 1993) using TnHind3 primer in the presence of 2 mM MgCl_2_. The initial denaturation step was carried out for 5 min at 94°C, followed by 30 cycles of 1 min denaturation at 94°C, 1 min annealing at 63°C and 2 min 50 s extension at 72°C, and a final extension step of 7 min at 72°C.

The complete *xer1* gene along with its putative promoter (978 bp) was amplified using PG2 genomic DNA and primers Xer1start_BamHI and Xer1stop_SmaI in the presence of 2.5 mM MgCl_2_. PCR cycling conditions consisted of an initial denaturation for 5 min at 94°C, 30 cycles of 94°C for 1 min, 53.5°C for 1 min and 72°C for 1 min, followed by 7 min at 72°C.

Detection of Tet^R^ and Gent^R^ determinants in picked transformants was carried out by PCR methods described previously (Chopra-Dewasthaly *et al*., 2005a,b) using primer pairs TetF/TetR and Tn1/Tn2 respectively.

### Construction of recombinant vectors

#### Plasmid pR2 (5.67 kb)

The approximate 0.5 kb partial x*er1* sequence was amplified (as described above) and digested with KpnI/XbaI and cloned into the same sites of pUC18 (Invitrogen). The resulting plasmid construct was then digested with HindIII and ligated to the 2.5 kb HindIII fragment of pISM2062 (Knudtson and Minion, 1993) which contains the Gent^R^ determinant for selection in mycoplasmas.

#### Plasmid pR3 (10 kb)

pR2 was digested with HincII/EcoRI and the partial *xer1* fragment was excised and purified following agarose gel electrophoresis and then ligated to SmaI/EcoRI-digested *M. agalactiae oriC* vector, pMM21-7 (Chopra-Dewasthaly *et al*., 2005a), to generate the replicative plasmid pR3.

#### Plasmid pR4 (9.7 kb)

The *xer1* amplicon (as described in the previous section) was digested with BamHI/SmaI and cloned into the corresponding sites of Tn*4001*mod carried by the vector pISM2062 (Knudtson and Minion, 1993). The BamHI and SmaI sites in Tn*4001*mod are located within the P_IN_ promoter of the transposase gene in one IS element resulting in an inactivated P_IN_ promoter that cannot read against a gene inserted in these cloning sites (Lyon *et al*., 1984; Knudtson and Minion, 1993). The *xer1* gene together with its putative promoter was inserted such that the transcription was directed outwards of the transposon. Thus, the existing P_OUT_ promoter of the IS element will drive transcription in the same direction as the putative *xer1* promoter rather than inhibiting transcription (Lyon *et al*., 1984; Waldo and Krause, 2006). The nucleotide sequence of *xer1* portion of pR4 was determined to ensure that no errors had been introduced during PCR amplification and cloning.

#### Plasmid p21-7Gxer (9.5 kb)

SacI-digested 6.013 kb fragment of pMM21-7 was gel extracted and ligated to the SacI-digested 2.5 kb fragment (carrying Gent^R^ determinant) obtained by PCR. The resultant *oriC* plasmid p21-7G was sequentially digested with BamHI/SmaI and ligated to the BamHI/SmaI-digested 978 bp *xer1* amplicon to produce p21-7Gxer.

### Transformation of *M. agalactiae*

Transformations were carried out essentially as earlier described (Chopra-Dewasthaly *et al*., 2005b). Mid-log-phase PG2 cells were electroporated with 2.5 μg of plasmid pR3 in a 2 mm electrocuvette (at 2.5 V, 25 μF and 100 Ω) to obtain *xer1* disruption mutants. After an initial growth of 2 h in non-selective SP-4 medium, 2 μg ml^−1^ tetracycline was added and cells were allowed to grow overnight. The transformation mix was passaged daily by a 1:10 dilution into 1 ml of fresh SP-4 broth for 10–15 days, whereby the tetracycline concentration was gradually increased from 2 to 10 μg ml^−1^. From passage 5 onwards the cells were checked for the presence of *xer1* disruption by PCR (explained above) and subjected to Southern blot hybridization. For complementation of the *xer1* mutation, PLMU was transformed with 2.5 μg of plasmid p21-7Gxer and grown for 2 h in non-selective liquid medium before adding 2 μg ml^−1^ tetracycline and 50 μg ml^−1^ gentamicin, followed by overnight incubation at 37°C and then plating on selective SP-4 agar the next day.

### Oligonucleotides and sequencing

DNA sequencing and the synthesis of all oligonucleotides used in this study (Table S1) were carried out at VBC-Biotech Services, Vienna. Sequences were analysed by advanced blastx searches (Altschul *et al*., 1997) made at the website for the National Center for Biotechnology Information (http://www.ncbi.nlm.nih.gov/blast/blast.cgi) using the mold mitochondrial genetic code.

### Prediction of Rho-independent terminator structures

Rho-independent terminator structures were predicted based on the method of de Hoon *et al*. (2005). Briefly, downstream regions of the *vpma* genes were searched for T-stretches with a minimum length of 14 nucleotides. Contrary to the method of de Hoon *et al*. (2005), we required the T stretch to start with at least four consecutive thymidine residues instead of two, as mycoplasmas have a higher AT DNA content than the bacteria dealt with by de Hoon *et al*. (2005). Calculation of the RNA secondary structure and the Gibbs free energy of formation for the terminator stem-loop structure were performed with Mfold (Zuker, 2003) at a temperature of 25°C as described (de Hoon *et al*., 2005).

The T-stretch score was evaluated as described by de Hoon *et al*. (2005):

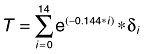

where *δ*_*i*_ is one if the *i*th nucleotide is a thymidine and zero otherwise.

The decision role *d* was calculated as described by de Hoon *et al*. (2005):

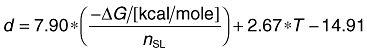

where Δ*G* is the Gibbs free energy of stem-loop formation in kcal/mole at a temperature of 25°C, *n*_SL_ is the number of nucleotides in the entire stem-loop structure and *T* denotes the T-stretch score described above. Values higher than zero (discriminant line *d* = 0) indicate terminator structures and higher *d* values correlate with stronger termination efficiency (d'Aubenton Carafa *et al*., 1990; de Hoon *et al*., 2005).

## Supplementary material

This material is available as part of the online article from: http://www.blackwell-synergy.com/doi/abs/10.1111/j.1365-2958.2007.06103.x

(This link will take you to the article abstract).
