# Supplementary material for: Phase-locked mutants of Mycoplasma agalactiae: defining the molecular switch of high-frequency Vpma antigenic variation
Source: Mol Microbiol. 2008 Jan 30;67(6):1196–210. doi: 10.1111/j.1365-2958.2007.06103.x (PMC2268961; doi:10.1111/j.1365-2958.2007.06103.x)
Supplement: Supplementary file 1 [file mmi0067-1196-SD1.pdf]

Supplementary Material

Phase-locked mutants of *Mycoplasma agalactiae*: Defining the molecular switch of high-frequency Vpma antigenic variation

Rohini Chopra-Dewasthaly[1*], Christine Citti[1,2], Michelle D. Glew[1,3], Martina Zimmermann[1], Renate Rosengarten[1] and Wolfgang Jechlinger[1†]

[1]Institute of Bacteriology, Mycology and Hygiene, Department of Pathobiology, University of Veterinary Medicine Vienna, Veterinärplatz 1, A-1210 Vienna, Austria

[2]Present address: UMR1225, INRA, ENVT, Ecole Nationale Vétérinaire, 23 Chemin des Capelles, BP 87614, F-31076 Toulouse Cedex 3, France

[3]Present address: Bio21 Institute, School of Dental Science, 30 Flemington Rd, Parkville 3010, Australia

[†]Deceased

*For correspondence: Phone: +43-1-25077-2105, Fax: +43-1-25077-2190, E-mail: Rohini.Chopra-Dewasthaly@vu-wien.ac.at

Supplementary Material

Table S1. Oligonucleotide sequences used in this study

Name	Sequence (5' to 3')[a]	Source/Reference
TnHind3	ATCGGAgagctcAGCTTGCGCATCATTGG	This study
Xer1start_*Bam*HI	GATGTAggatccATTTATGATAAGTTATGC	This study
Xer1stop_*Sma*I	ATATTAcccgggATTCTACTTACTATTAAG	This study
RecendET28	GACGAGaagcttACTATTAAGCATTATTTTTC	This study
T3ISLrev	AGAGCAgaattcAATTAACCCTCACTACTAAAG	Chopra-Dewasthaly *et al*, 2005b
TetF	CATGTGGAGATAGAAC	Chopra-Dewasthaly *et al*, 2005b
TetR	GATATTCCTGTGGCGC	Chopra-Dewasthaly *et al*, 2005b
Xer-S	GCTAGGtctagaTAGAGTGATATACGACAC	This study
Xer-R	TACTGTggtaccTAGACTATTGATGCTTAC	This study
Xerloc	GGTTTCTACCATATTGACTCC	This study
Tn1	ACATGAATTACACGAGGGC	Chopra-Dewasthaly *et al*, 2005a
Tn2	GTTCTTCTTCTGACATAGTAG	Chopra-Dewasthaly *et al,* 2005a
Z2F	CGCggatccGTTGCACAAACAGATTCCGAC	This study
Z1R	AAActgcagTTATTCGTATTTAGGTAATAGTCTTC	Glew *et al*, 2002
X1F	CGCggatccAAAGTAATGAAGGTCAATTACC	Glew *et al*, 2002
X1R	AAActgcagGCTTAAGGATTTTTTAAAATGATG	Glew *et al*, 2002
C1F	CCGgaattcGTTGAGGAAGCAATTAAAACAGC	This study
C1R	GCtctagaTTATCCAGATGATGTTTCAACTTC	This study
D1F	CGCggatccAATGGCGGAAATAGTAATGGTAAC	This study
D1bR	AAActgcagAATAACTTTATCTAGTTCTATACC	This study
U2F	CGCggatccGATAAAGAAGATAAGACAGGTG	This study
U2R	AAActgcagTTAACTTGATTCCATTGGGACACT	This study
Y3F	CCGgaattcAATGCAAACGCTGCAGAAAATG	This study
Y3R	GCtctagaTTAAGTAAATGTAACTGTAACTTCACC	This study

[a]Lower case letters represent restriction sites introduced to enable cloning.

Supplementary figure 1

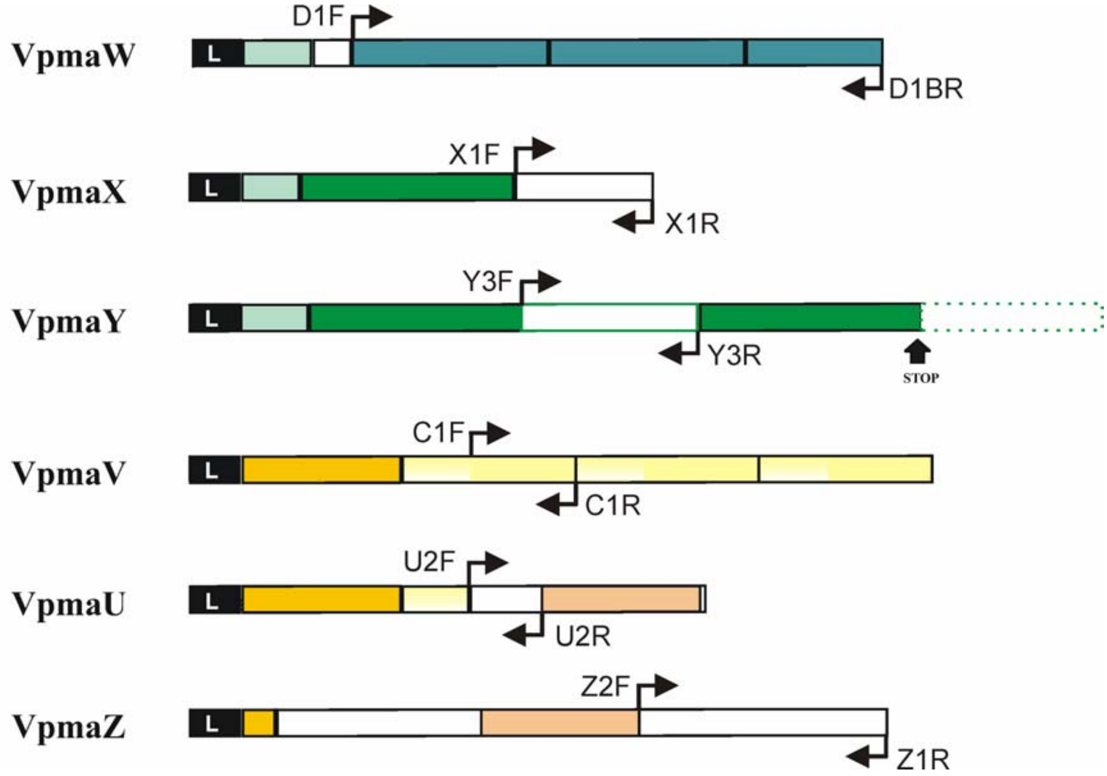

Fig. S1. Schematic drawing of the structure of the six Vpma proteins depicting the regions which were incorporated into MBP fusion proteins (adapted from Glew *et al.*, 2002) for the production of polyclonal antibodies. These regions are exclusively present in the respective Vpma protein and are indicated by flanking arrows together with the name of the primers used for amplification (see Table S1). Homologous or repeated regions (homologies ranging from 61 to 100%) are indicated in the same colours, whereas regions being unique are white (Glew *et al.*, 2002). The white region with a green contour in the VpmaY protein is unique, but the encoded sequence is identical to that of an untranslated reminder of the VpmaY repeat indicated by a dotted green line. Each Vpma protein starts with a homologous 25 bp leader sequence (L).

Supplementary figure 2

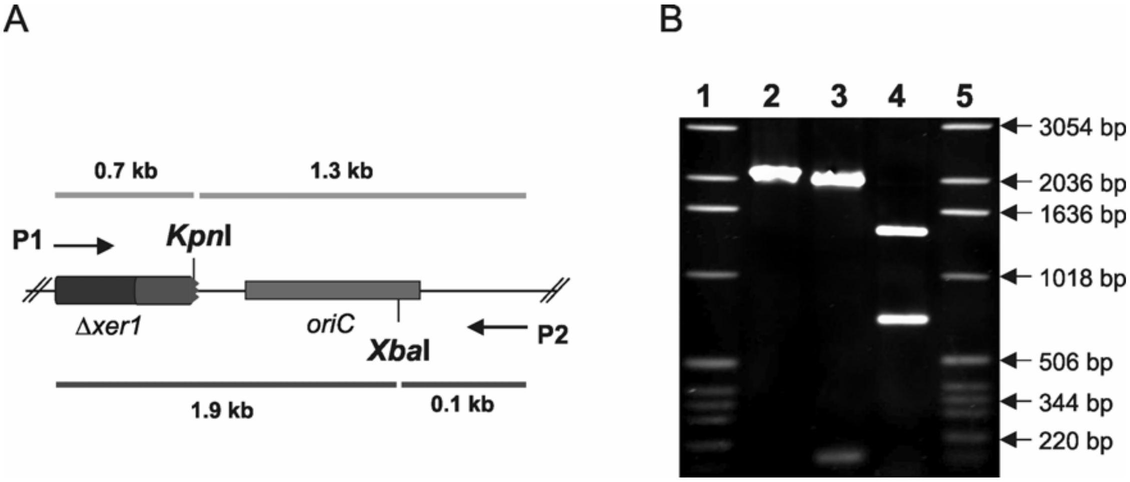

Fig. S2. Detection of *xer1* disruption in *M. agalactiae* type strain PG2 by PCR and restriction analysis of the amplicon specific for the integrated pR3 plasmid. (A) Schematic representation of the binding sites of the primers RecEndET28 (P1) and T3ISLrev (P2) specific to the chromosomal *xer1* region and the pR3 plasmid backbone, respectively, in the genomic DNA of a *xer1* mutant. The expected sizes of the restriction fragments obtained after *Kpn*I and *Xba*I digestions of the 2 kb PCR product are indicated. (B) Agarose gel analysis of PCR product: (lane 2) uncut, (lane 3) *Xba*I-digested, (lane 4) *Kpn*I-digested. Lanes 1 and 5 represent the λ-*Hin*dIII DNA size marker.

Supplementary figure 3

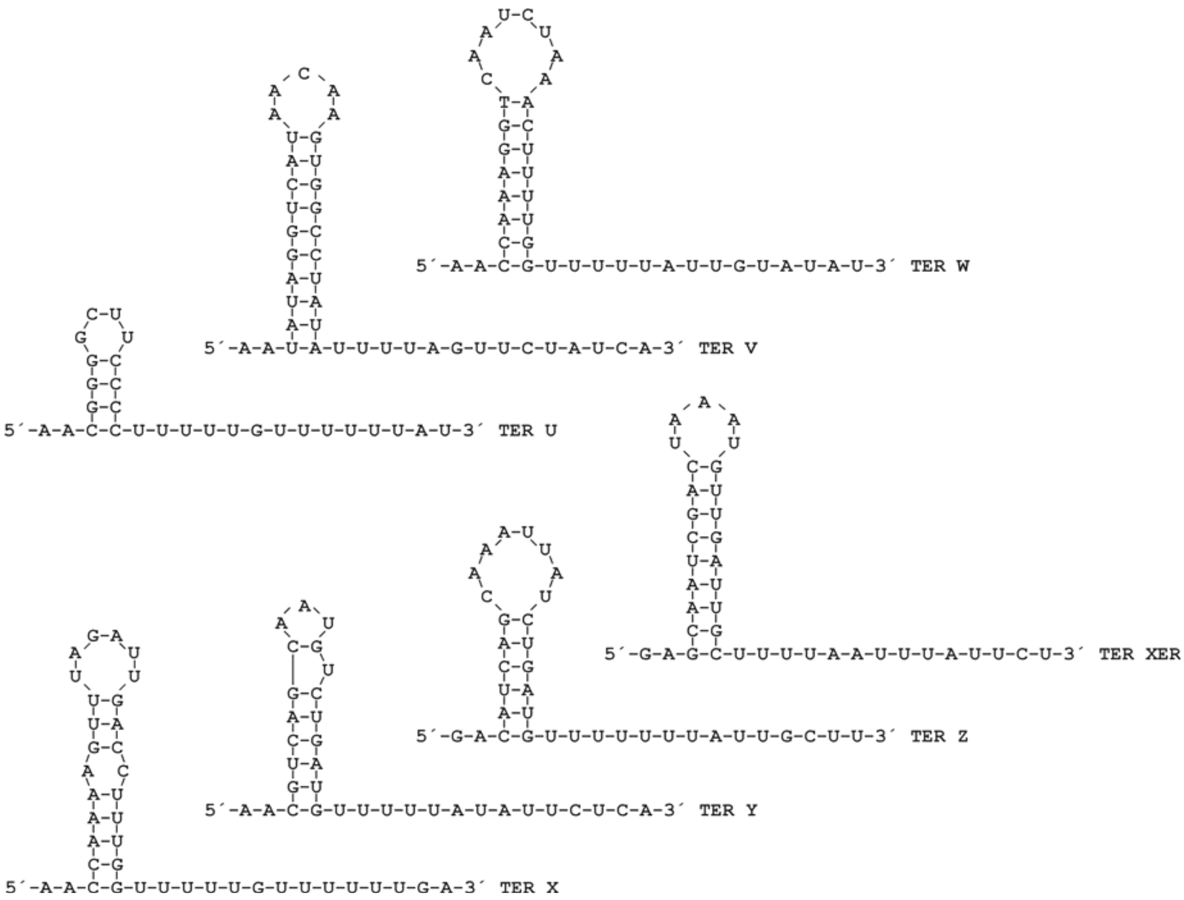

Fig. S3. Rho-independent terminator structures in the *vpma* locus. RNA secondary structures derived from the primary DNA sequence and from the RNA-fold prediction program Mfold (Zuker, 2003).

Supplementary figure 4

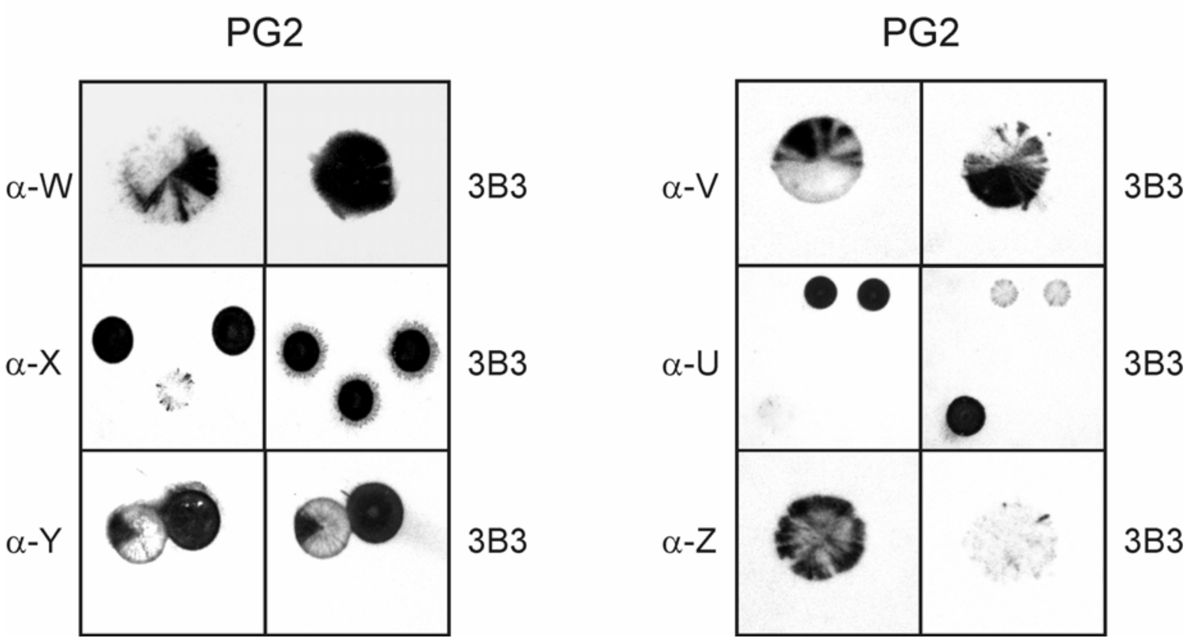

Fig. S4. Comparative immunoblot staining of *M. agalactiae* type strain PG2 using MAb 3B3 (Bergonier *et al.*, 1996) and monospecific PAbs generated in this study. The same colonies were lifted twice on separate nitrocellulose membranes, whereby the first lift was exposed to the respective Vpma-specific PAb (left columns) and the second to MAb 3B3 (right columns). The MAb 3B3 epitope was reported to be carried by several proteins (Bergonier *et al.*, 1996), and though one of these was shown to be Vpma Y (Glew *et al.*, 2000, Glew *et al.*, 2002), we suspected the involvement of more Vpmas. **A:** MAb 3B3 indeed recognized three different Vpma proteins, namely VpmaW, VpmaX and VpmaY, all of which share large regions of homology (Glew *et al.*, 2002, Fig. S1). **B:** Similarly, VpmaV, VpmaU and VpmaZ, share homologous regions and none of these Vpmas bear MAb 3B3 epitopes. This result indicates that the actual frequency of Vpma switching is much higher than the earlier estimates based on MAb 3B3.
